# Comparative analysis of serine protease-related genes in the honey bee genome: possible involvement in embryonic development and innate immunity

**DOI:** 10.1111/j.1365-2583.2006.00684.x

**Published:** 2006-10

**Authors:** Z Zou, Dawn L Lopez, Michael R Kanost, Jay D Evans, Haobo Jiang

**Affiliations:** * Department of Entomology and Plant Pathology, Oklahoma State University Stillwater, USA; † Department of Biochemistry, Kansas State University Manhattan, USA; ‡ USDA-ARS Bee Research Laboratory Beltsville, USA

**Keywords:** *Apis mellifera*, insect immunity, serine protease homolog, serpin, clip domain, phylogenetic analysis, protease cascade

## Abstract

We have identified 44 serine protease (SP) and 13 serine protease homolog (SPH) genes in the genome of *Apis mellifera*. Most of these genes encode putative secreted proteins, but four SPs and three SPHs may associate with the plasma membrane via a transmembrane region. Clip domains represent the most abundant non-catalytic structural units in these SP-like proteins −12 SPs and six SPHs contain at least one clip domain. Some of the family members contain other modules for protein–protein interactions, including disulphide-stabilized structures (LDL_r_A, SRCR, frizzled, kringle, Sushi, Wonton and Pan/apple), carbohydrate-recognition domains (C-type lectin and chitin-binding), and other modules (such as zinc finger, CUB, coiled coil and Sina). Comparison of the sequences with those from *Drosophila* led to a proposed SP pathway for establishing the dorsoventral axis of honey bee embryos. Multiple sequence alignments revealed evolutionary relationships of honey bee SPs and SPHs with those in *Drosophila melanogaster*, *Anopheles gambiae*, and *Manduca sexta*. We identified homologs of *D. melanogaster* persephone, *M. sexta* HP14, PAP-1 and SPH-1. *A. mellifera* genome includes at least five genes for potential SP inhibitors (serpin-1 through -5) and three genes of SP putative substrates (prophenoloxidase, spätzle-1 and spätzle-2). Quantitative RT-PCR analyses showed an elevation in the mRNA levels of SP2, SP3, SP9, SP10, SPH41, SPH42, SP49, serpin-2, serpin-4, serpin-5, and spätzle-2 in adults after a microbial challenge. The SP41 and SP6 transcripts significantly increased after an injection of *Paenibacillus larva*, but there was no such increase after injection of saline or *Escherichia coli*. mRNA levels of most SPs and serpins significantly increased by 48 h after the pathogen infection in 1st instar larvae. On the contrary, SP1, SP3, SP19 and serpin-5 transcript levels reduced. These results, taken together, provide a framework for designing experimental studies of the roles of SPs and related proteins in embryonic development and immune responses of *A. mellifera*.

## Introduction

Serine proteases in the S1 family (e.g. chymotrypsin) are involved in various physiological processes, such as digestion, development, and defense responses (Rawlings & Barrett, 1993; Krem & Di Cera, 2002). They are typically synthesized as zymogens, which require proteolysis at a specific site for activation. In some cases, after an initiation protease becomes active upon stimulation, other downstream SP zymogens are sequentially activated in a cascade pathway, which eventually generates effector molecules by limited proteolysis. High specificity of their catalytic domains, interactions among the regulatory regions, and efficient removal of active SPs by irreversible protease inhibitors ensure local, transient reactions to physiological or pathological cues. Human blood coagulation and complement activation are the best known examples of such protease systems (O’Brien & McVey, 1993; Whaley & Lemercier, 1993). The evolutionary history of serine protease pathways can be traced back to the divergence of deuterostomes and arthropods (Iwanaga *et al*., 1998; Jiang & Kanost, 2000; Krem & Di Cera, 2002; Kanost *et al.*, 2004). Recently, biochemical and genomic analyses revealed that catalytically inactive serine protease homologs are also constituents of these systems (Kwon *et al*., 2000; Yu *et al*., 2003). SPHs are similar in sequence to S1 proteases but lack one or more of the catalytic residues in SPs. A human SPH named azurocidin mediates inflammation and has an antimicrobial activity (Watorek, 2003). Invertebrate SPHs participate in acute-phase responses (Kawabata *et al*., 1996; Huang *et al*., 2000; Yu *et al*., 2003).

The horseshoe crab haemolymph clotting system represents the best characterized SP system in invertebrates (Iwanaga *et al*., 1998). It is composed of four proteases (Factors C, G, B, and clotting enzyme) and one clottable protein (coagulogen). In *Drosophila*, genetic studies revealed a SP pathway that establishes the dorsoventral axis of embryos (Belvin & Anderson, 1996). This pathway also comprises four proteases, namely nudel, gastrulation defective, Snake, and easter. Easter cleaves spätzle to form an active ligand that binds to the Toll receptor and triggers the intracellular signalling pathway for ventralization. In *Drosophila* adults, another set of SPs leads to spätzle activation and drosomycin production (Lemaitre *et al*., 1996). Another insect defense mechanism involving a SP cascade is the proteolytic activation of prophenoloxidase (proPO) (Ashida & Brey, 1998; Ligoxygakis *et al*., 2002b; Kanost *et al*., 2004). In *Manduca sexta*, HP14 and proPO-activating proteases (PAPs) are the first and last components of the proPO activation cascade (Ji *et al*., 2004; Jiang *et al*., 1998; Jiang *et al.*, 2003a and 2003b; Lee *et al*., 1998; Satoh *et al*., 1999). Our knowledge on composition, order, and regulation of these insect SP cascades has greatly expanded (Levashina *et al*., 1999; Ligoxygakis *et al*., 2002a; Kim *et al*., 2002; Gupta *et al*., 2004; Tong *et al*., 2005; Zou & Jiang 2005; Jiang *et al*., 2005; Wang *et al*., 2006; Wang & Jiang, 2004 and 2006; Jang *et al*., 2006).

Genome-wide analyses of SPs and SPHs are available for *Drosophila melanogaster* and *Anopheles gambiae* (Christophides *et al*. 2002; Ross *et al*., 2003). However, little is known about these proteins in the honey bee. Among ∼1.0 × 10^4^ predicted genes in the genome of *A. mellifera*, SP and SPH genes form a large family (Honey Bee Genome Sequencing Consortium, 2006; Evans *et al*., 2006). To begin to understand the potential functions of SPs in immune responses in this beneficial insect, it is necessary to annotate these genes, compare their protein products with homologous molecules from other insects, and predict their functions. In this paper, we report a genome-wide analysis of the structures, evolutionary relationships, and possible physiological functions of *A. mellifera* SPs and SPHs. Some putative substrates and inhibitors of SPs are also discussed. We hope that these results could provide evolutionary perspectives of the S1 family of protease genes in insects and stimulate interest for in-depth analyses of SP-related proteins (i.e. SPs, SPHs, serpins and SP substrates) in the honey bee.

## Results and discussion

### Overview of the SP-SPH gene family

Blast searches of the *A. mellifera* genome yielded 57 sequences with significant similarity to the S1 protease family. Compared with 204 in *D. melanogaster* (Ross *et al*., 2003) and 305 in *An. gambiae* (Christophides *et al*. 2002), the number of SP-like genes in the honey bee is much smaller. We retrieved and annotated the sequences from Official Gene Set-1 (Honey Bee Genome Sequencing Consortium, 2006). Based on the presence or absence of residues essential for the catalytic activity of SPs, we classify them as SPs or SPHs. We identified 44 SP and 13 SPH genes in the bee genome (Table 1). The ratio of SPs to SPHs is close to that in *D. melanogaster*, which has 147 SPs and 57 SPHs. *A. mellifera* SP11, SP29, SPH50 and SPH51 are clustered in Group 9.19–20; SP4, SP5, SP8, SP13 and SP27 in Group 15.3–8; SP25, SP33 and SPH56 in Group 13.1–3. The other genes are widely spread over the genome. In contrast, large clusters of SP/SPH genes are common in the genomes of *D. melanogaster* and *An. gambiae*. It appears that this gene family may have undergone a major expansion in the Diptera that did not occur in Hymenoptera after divergence of these orders more than 240 million years ago.

**Table 1:** Serine proteases (SPs) and serine proteinase homologs (SPHs) in *Apis mellifera*

		Homologous proteins		Conserved regions^b^						
							
Gene name	ID	Drosophila	other arthropods^a^	TAAHC	DIAL	GDSGGP	Length^c^ (aa)	Activation site^d^	Enzyme specificity^e^	Domain structure^f^
cSP1	16147	ea CG1102	MsHP5				376	TEQK∧IFGG	T(DGA)	C-SP
cSP2	14247	ea CG1102	MsHP8				∼391	LSQR∧IIGG	?(D??)	C-SP
cSP3	11698		MsHP17				353	SHTR∧VVGG	T(DGG)	C-SP
SP4	10646	CG4914					> 304	EESR∧IVGG	T(DGG)	SP
SP5	12300	CG4386 CG18735					329	VQRR∧IVGG	T(DGG)	SP
cSP6	14077	CG8172			DVAL		622	RSNR∧IVGG	T(DGG)	2LC-C-SP
cSP7	17145	CG31728					512	DQER∧IVGG	T(DGG)	C-SP
SP8	18767	CG9372	MsHP21		DIAV		> 292	SRSR∧LTGG	T(DGG)	SP
cSP9	18732	CG11843	MsHP13				423	DRKL∧IVGG	T(DGG)	pSP-SP
cSP10	17927	Psh	MsHP21 CfSP				?751	PNKF∧IVGG	T(DGG)	2(C-LC-SP)
		Psh	MsHP21 CfSP					PNKF∧IVGG	T(DGG)	
SP11	14654	CG11836	MsHP1		DVAL		> 255	QEDR∧IVGG	T(DGG)	SP
SP12	19856	CG5255 CG31265			DIGL		> 237	EIPK∧IVGG	?(GGD)	SP
SP13	15640	CG7996	MsHP21 Ag18D				> 448	PNHL∧VIGG	T(DGG)	3LC(Nr)-SP
cSP14	14044	CG2056-PB,snake	MsHP6		DVAI		> 385	LSFH∧IFNG	T(DGG)	C-SP
SP15	18178						> 294	TTGR∧IFNG	?(GGD)	SP
SP16	12253	CG16996		TAGHC	DLAL		?1149	PETR∧IVGG	T(DGG)	2LC(HTr)-SP
SP17	14603	CG4316		TAGHC			?498	LEPR∧ITGD	C(SAG)	SP-SP
		CG4316		TAGHC				FDTR∧IVGG	C(SGS)	
SP18	10222	CG31954	CsSP		DVAL		> 247	LQPR∧IIGG	T(DGG)	SP
cSPH19	17345	CG4998	TtFD		DIAI	GDGGGP	741			4LC-LC(Yr)-C?-SPH
SP20	19590	nudel corin	BmOvarianSP		DIGM		?1645	SQLR∧VVGG	T(DGG)	3LDLA-SP-3LDLA-pSP-LDLA(RGD)-2LDLA(pSP)
cSP21	16220	CG7432	TtPCE		DIAV		> 408	GKYR∧VVGG	T(DGG)	C-LC-SP
SP22	13791		CsSP				> 259	PDTQ∧IVGG	T(DGG)	SP
SP23	12538	Tequila CG4821	Ag22D				?2323	IFQK∧VVRG	T(DGG)	4LC-4CBD-SR-Clect-KR-LDLA-PA-2LDLA-SP
SP24	14233	CG6865			DIAI		> 236	?	T(DGG)	SP
cSP25	19719	CG11824			DLAL		?942	PESR∧IVGG	T(DGG)	C-10LC(STr)-SP
cSP26	18450	CG8170	CfSP	TAGHC	DVAV		?667	AQRR∧IVGG	T(DGG)	TM-2LC-C-SP
SP27	11588	CG31954	AgTry				?537	MDGR∧IVGG	T(DGG)	2(LC-SP)
					DVAV			PTGQ∧IIGG	T(DGG)	
SP28	13489	CG30375			DVAL	MDSGGP	> 405	NPSR∧IVGG	T(DGG)	TM-CUB-SP
SP29	14644	CG18375			DIAI		> 224	?	T(DGG)	SP
SP30	19649	corin		TASHC	DVAL		∼944	AKTR∧IVGG	T(DGG)	cc-LC-TM-Fri(ZnF)-LDLA-LC-LDLA-SR-SP
SP31	11297		AaTry	TAGHC			> 291	EEDR∧IFGG	T(DGG)	SP
SP32	11511		AaChy	TAGHC			> 260	RPTR∧IVGG	C(AGS)	SP
cSP33	14309	CG8213	OnT2b		DLAL		> 1269	KSGR∧IVGG	T(DGG)	C-5LC(Tr)-SP
SP34	11552	CG30371	CsChy	TAGHC		MGSGGP	∼405	NPSR∧IVGG	?(DGN)	TM-CUB-SP
SP35	16021	CG5255			DIAI		> 255	NLEK∧IVGG	?(GVD)	LC-SP
SP36	19846	CG5255					> 263	PESK∧IVGG	?(GGD)	SP
SPH37	18944	CG13318	PlMasq	TVAHC	DVAV	GDGGSP	> 307			pC?-SPH
SP38	16214	CG10663			DVAM		∼481	YFTR∧IIGG	T(DGG)	2TSP1-SP
cSPH39	14366	LD13269p	CrVn50 TmPPAF		DIAV	GDGGGP	?783			ZnF-LC-C-LC(Tr)-C-C(LC)-C-SPH
SP40	13263	CG32808	PlTry				?725	YNPK∧IING	C(GAT)	ZnF-LC-Sina-LC-SP
cSPH41	10943	masquerade CG15002				GDGGGP	735			5[C-LC(STr)]-SPH
cSPH42	11298	CG5390	BmMasq CrVn50		DFAI	GDGGSP	417			LC-C-SPH
SP43	18530	CG9564			DVAV		268	PTGQ∧IIGG	T(DGG)	SP
SP44	15453				DITI	GDSGGG	> 340	LIGR∧IVNG	T(DGG)	SP
SP45	17654			SAAHC	DIAM		1748	RRSR∧IVGG	?(D??)	TM?-6LC-3LDLA-SP
SP46	16367	CG13461 stubble gd	MsHP19		DLAV	GDSGSG	439	FNLL∧VAGG	E(GSI)	SP
SP47	14774			-----			> 157	?	?(DI?)	pSP
SP48	12379	CG32376	MsHP3	TALHC			> 257	ATIK∧IIGG	T(DGG)	SP
SP49	15317	CG31217	MsHP14		DIAI	GDSGGG	∼628	SKTL∧IVNG	E(SSS)	LC-4LDLA-Sushi-Wonton-SP
cSPH50	14001	CG14945	MsPAP1	TTANC	NIAM	GYNGSP	707			TM-LC-PLCXc-C-SPH
SPH51	13397	CG18735 CG4386		TCGNC	----	LDVSSS	> 296			SPH
SPH52	19292			-----	----		> 136			pSP
SPH53	15702			TSAQC	NIAL	GNPGSP	> 294			LC-SPH
SPH54	15980			ASYSC		NDEGAP	?2733			TM-LC-EGF-13LC(HEPSr)-5LDLA-SR-SPH
cSPH55	15254	CG11066		TAANC	DLAT	TDIGSP	> 539			C-SPH
SPH56	13019	CG1632		TTASC	TTVL	EFAGSP	∼777			LC-TM-SEA-LC-FRI-2LDLA-SPH
SPH57	16038	CG31954		-----		------	> 159			pSPH

a Aa, *Aedes aegypti*; Ag, *Anopheles gambiae*; Bm, *Bombyx mori*; Cf, *Ctenocephalides felis*; Cr, *Cotesia rubecula*; Cs, *Culicoides sonorensis*; Ms, *Manduca sexta*; On, *Ostrinia nubilalis*; Pl, *Pacifastacus leniusculus*; Tm, *Tenebrio molitor*; Tt, *Tachypleus tridentatus*.

b If not listed, sequences are identical to the conserved TAAHC, DIAL, or GDSGGP. -----: conserved region not identified.

c >, incomplete sequence due to prediction errors; ∼, nearly complete (e.g. partial signal peptide); ?, prediction error?

d ∧, putative activation cleavage site; ?, not predicted; blank, not applicable (SPH).

e Enzyme specificity predicted based on Perona and Craik (1995). T, trypsin; C, chymotrypsin; E, elastase; ?: not predictable; blank: not applicable (SPH). Letters in parentheses: amino acid residues determining the primary specificity of a serine proteinase.

f C, clip domain; CBD: chitin-binding domain; cc, coiled coil region; Clect, C-type lectin domain; CUB, a domain identified in **C**omplement 1r/s, **U**egf, and **B**mp1; EGF, ^Ca2+^-binding EGF domain; FRI, frizzle domain; KR: kringle domain; LC, low complexity region; LDLA: low-density lipoprotein receptor class A domain; p, partial; PA, pan-apple domain; PLCXc, phospho-lipase C catalytic domain; SEA, a ∼120-residue domain in **S**perm protein, **E**nterokinase and **A**grin; Sina, a domain identified in *Drosophila****s****even****in a****bsentia*; SP, serine proteinase catalytic domain; SPH, serine proteinase-like domain; SR: **s**cavenger **r**eceptor cysteine-rich domain; Sushi, Sushi domain, also known as CCP or SCR. Wonton: a disulfide knotted domain found in *M. sexta* HP14; TSP1, thrombospondin type I repeat; TM, transmembrane region; XYr, regions rich in amino acid residues X and Y; ZnF, Zinc finger domain.

The catalytic triad of S1 proteases is composed of His^57^, Asp^102^ and Ser^195^ (chymotrypsin numbering). In most cases, these residues are present in highly conserved sequence motifs of TAA**H**C, **D**IAL and GD**S**GGP (Table 1). One or more of the catalytic residues are replaced in SPHs. GD**S**GGP is present in 32 of the honey bee SPs. In the 13 SPHs, 5 contain GDGG in the context of GDGGGP or GDGGSP. His^57^, also critical for protease activity, is located in TAA**H**C or its analogs: TAA**H**C and TAG**H**C are present in 67% and 12% of the SP/SPH family members, respectively. Asp^102^, the 3rd member of the catalytic site, is located in **D**IAL (28), **D**VAL (5), **D**VAV (4), **D**IAI (4), **D**LAL (3) or **D**IAV (3), where the number in parentheses indicates its occurrence in the SP-like sequences. While most SPs or SPHs are expected to be extracellular proteins, we only found 13 with a complete signal peptide for secretion. The gene prediction programs apparently failed to locate exons encoding such short sequences, which lack particular structural features other than having a stretch of hydrophobic residues.

### Single domain SPs

Digestive SPs (e.g. trypsin) have a relatively simple structure, containing ∼240 residues. Fourteen *A. mellifera* SPs, shorter than 300 residues, may function in digestion, a process that does not require sophisticated protein–protein interactions. The bee has far fewer single domain SPs compared with ∼80 in *D. melanogaster* and ∼140 in *An. gambiae.* This could be related to its relatively simple food source, nectar and pollen. Nearly all of these putative digestive proteases reside in one branch of the honey bee SP-SPH phylogenetic tree, representing descendents of a simple ancestral SP gene (data not shown). On the other hand, 39 (or 69%) of the *A. mellifera* SPs and SPHs are longer than 300 residues. Only 1/2 and 1/3 of the family members in *D. melanogaster* and *An. gambiae* may contain additional regulatory domains. These proteins are probably involved in more complex physiological processes in which other structural units are needed for molecular recognition.

### Clip-domain SPs and SPHs

In arthropods, clip-domain SPs mediate innate immunity and embryonic development (Jiang & Kanost, 2000; Kanost & Clarke, 2005). Each clip domain contains three disulphide bonds, and many SPs and SPHs between 300 and 400 residues contain one such domain. Although clip domain sequences are hypervariable, we have identified 12 cSPs and six cSPHs in the honey bee by locating the conserved pattern of Cys residues. Consistent with the small overall family size, the total number of *A. mellifera* cSPs and cSPHs is ∼1/3 of that in the *Drosophila* or *Anopheles*. In the bee, we did not find any dual clip-domain SPs, which serve as PAPs in *M. sexta* and *Bombyx mori* (Satoh *et al*., 1999; Jiang *et al*., 2003a and 2003b).

**Figure 1:**
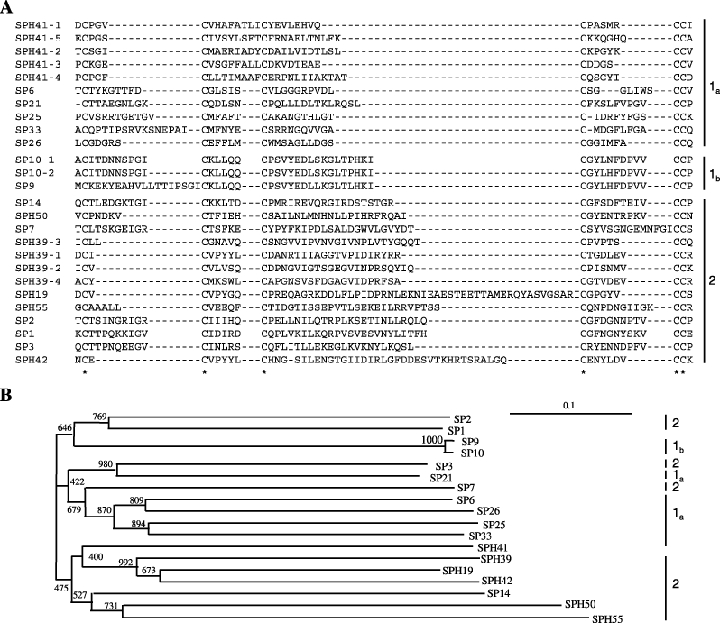
Sequence comparison and phylogenetic relationships among the *Apis mellifera* clip-domain SPs and SPHs. A. alignment of the clip domain sequences. Six conserved Cys residues form 3 disulphide bonds. B. phylogenetic tree based on an alignment of the catalytic and protease-like domains. Vertical bars and numbers indicate the clip domain groups.

The clip domains in *A. mellifera* SPs/SPHs range from 30 to 70 residues between Cys_1_ and Cys_6_, with an average size of 45 residues (Fig. 1A). The regions between Cys_2_ and Cys_3_ are exactly five residues, except for cSPH41. The lengths between Cys_3_ and Cys_4_ of cSPs are similar to those in cSPHs. According to our previous analyses (Jiang & Kanost, 2000; Ross *et al*., 2003), clip domains can be divided into two groups based on the number of residues between Cys_3_ and Cys_4_. Group 1 contains less than 16 residues whereas Group 2 is longer (average size: ∼23 residues). All Group 1_a_ cSPs in the honey bee are predicted to be activated by proteolytic cleavage between Arg and Ile (Table 1). They form one clade in the phylogenetic tree (Fig. 1B), except for SP7. In Group 1_b_, the Arg residue before the scissile bond is replaced by Phe or Leu. The corresponding position is occupied by Arg in Group 2 SPs, except for cSP14 – cSP14 is probably cut after a His residue, and it lacks the signature Cys pair present in most Group 2 cSPs (Ross *et al*., 2003).

A multiple sequence alignment of their catalytic domains suggests that all of the cSPs have a trypsin-like specificity, based on residues predicted to form the primary substrate-binding site (Table 1). A highly conserved Cys after the active site Asp in the context of PICLP is predicted to form a disulphide bond with a Cys in the linker between the clip and catalytic domains (based on horseshoe crab clotting enzyme). The phylogenetic analysis also indicates that clip-domain SPs and SPHs are more closely related to each other than to other members of the family. The divergence of *A. mellifera* clip-domain proteins was apparently an early evolutionary event with no shuffling of clip and protease domains thereafter. Moreover, since members of the each subgroup (group-1_a_, −1_b_ or −2) are clustered with each other, they may represent the three lineages emerged from ancient splits of the gene family.

We identified putative *Drosophila* orthologs for many *A. mellifera* clip-domain proteins (Table 1). cSP10 has a four-domain structure of clip-catalytic-clip-catalytic, and both halves of the molecule are highly similar to *Drosophila* persephone. Persephone is a component of the fungal-responsive branch of the SP system that triggers the Toll pathway for induced synthesis of drosomycin (Ligoxygakis *et al*., 2002a). *A. mellifera* SP17 and SP20 also contain more than one catalytic domain. Further analyses are needed to verify whether these three genes indeed encode proteins with such unusual domain structures. *A. mellifera* cSP14 and cSP2, most similar to *Drosophila* Snake and easter, may participate in the early development of honey bee embryos. All of the cSPHs are located in one clade of the phylogenetic tree (Fig. 1B). cSPH39 contains 4 clip domains, and cSPH41, a homolog of *Drosophila* masquerade, has 5 clip domains.

### SPs and SPHs with complex domain structures

**Figure 2:**
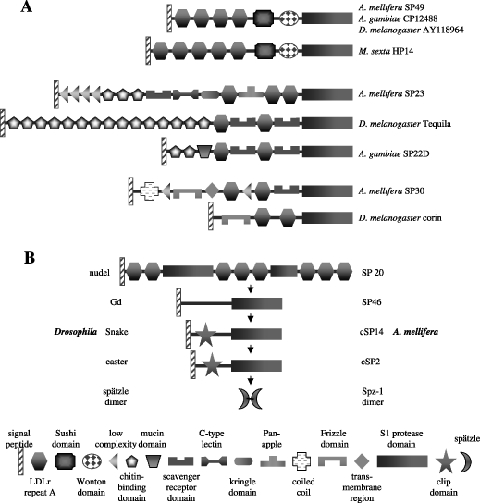
Domain organization of some SPs in *Apis mellifera* and other insects. A. *Apis mellifera* SP49 is orthologous to *Manducta sexta* HP14, *An. gambiae* AgCP12488 and *Drosophila melanogaster* AY118964. *Apis mellifera* SP23 is similar to *Anopheles gambiae* SP22D and *D. melanogaster* Tequila, whereas honey bee SP30 is homologous to *D. melanogaster* corin. B. A proposed SP cascade (left) for establishing the dorsal-ventral axis of *A. mellifera* embryo, in comparison to a similar system discovered in *D. melanogaster*.

Many of the SP/SPH family members contain other structural modules predicted to function in protein–protein interactions. These include several types of disulphide-stabilized domains (e.g. LDL_r_A, SRCR, frizzled, kringle, Sushi, Wonton and Pan/apple), carbohydrate-recognition domains (C-type lectin, chitin-binding), and other domains (e.g. zinc finger, CUB, coiled coil, and Sina) (Table 1 and Fig. 2A). SP20, SP23, SP30, SP45, SP49 and SP54 contain LDL_r_A repeats, which are ∼40-residue-long Cys-rich sequences first identified in the ligand-binding domain of low-density lipoprotein receptor (LDL_r_). SP23 is most similar to *An. gambiae* SP22D (Danielli *et al*., 2000; Gorman *et al*., 2000), but also resemble *D. melanogaster* Tequila in domain architecture (Fig. 2). Tequila has 15 chitin-binding domains, two scavenger receptor Cys-rich (SRCR) domains, 2 LDL_r_ Cys-rich domains and one SP domain (Munier *et al*., 2004). It also contains *His*- and *Pro*-rich regions and NGGYQPP repeats. At least three spliced forms of Tequila are detected throughout *Drosophila* development. Although there was no phenotype in the null mutant, its up-regulation in the wild-type fly upon fungal or bacterial infection suggests a role in innate immunity. In the mosquito, SP22D binds to chitin but not bacteria. The functions of *A. mellifera* SP23 and its orthologs in the fly and mosquito are unclear. *A. mellifera* SP49 is orthologous to *M. sexta* HP14, *An. gambiae* CP12488 and *D. melanogaster* AY118964 (Ji *et al*., 2004). These mosaic proteases have an identical domain structure: 4–5 LDL_r_A repeats, a Sushi domain, a Wonton domain and a SP catalytic domain (Wang & Jiang, 2006). *M. sexta* HP14 is an initiation enzyme activated upon pathogen recognition, and it triggers the SP pathway for proPO activation. *A. mellifera* SP49 may have the same function.

*A. mellifera* cSPH41 is orthologous to *Drosophila* masquerade, which is essential in the development of embryonic nerve tissues (Murugasu-Oei *et al*., 1995). SPH39 is identical in domain structure to *Drosophila* LD13269p (Table 1). SP30 and *Drosophila* corin are apparent orthologs, both containing a frizzle domain, LDL_r_A repeats and a type II transmembrane region (Fig. 2A). *A. mellifera* SP46 is an ortholog of *Drosophila* Stubble, a transmembrane SP required for leg and wing morphogenesis, which functions through a RhoA intracellular signalling pathway (Bayer *et al*., 2003).

### SP-mediated extracellular signal transduction

**Figure 3:**
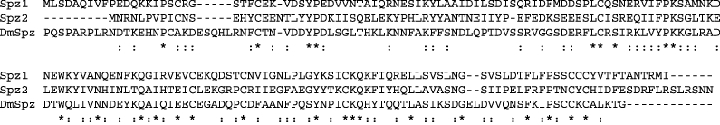
Alignment of *Drosophila* spätzle and Apis spätzle-1 and −2. The first 127 residues at the amino terminus of the fly protein were not shown. *, identical:, similar.

Formation of SP pathways is a common strategy employed by animals to respond to physiological or pathological stimuli. Genetic and biochemical analyses of protease cascades in model insects (e.g. *D. melanogaster*), when combined with genome sequences, may provide useful insights on similar processes in other arthropod species. Therefore, we compared the SP genes in the honey bee genome with Nudel, gastrulation defective (Gd), Snake, and easter, which establish the dorsoventral axis of *Drosophila* embryo (Belvin & Anderson, 1996). *A. mellifera* SP20 and SP46 are orthologous to Nudel and Gd, respectively (Fig. 2B). While high sequence similarity (identity: 26% and 39%) and identical domain structure suggest cSP14 and cSP2 may be honey bee Snake and easter, respectively, we are unable to assign unambiguous orthologous relationships due to the existence of other *Apis* clip-domain SPs with the same domain structure. Future experiments are needed to test whether *A. mellifera* SP20, SP46, cSP14 and cSP2 are involved in the early embryonic development. We have identified possible substrates for this proposed SP pathway, namely spätzle-1 (GB15688) and spätzle-2 (GB13503). *A. mellifera* spätzle-1 and −2 are 47% and 40% similar in sequence (identities: 28% and 22%) to *Drosophila* spätzle (Fig. 3). The numbers and positions of their Cys residues are conserved in most cases.

Proteolytic activation of proPO is a common defense mechanism in insects and crustaceans (Ashida & Brey, 1998). Active PO is involved in melanotic encapsulation and wound healing. In the last decade, this SP pathway has been extensively studied in *B. mori*, *M. sexta* and *Holotrichia diomphalia*. As described above, *A. mellifera* SP23, the ortholog of *M. sexta* HP14, may be an initiation protease of the pathway. While intermediate steps of the cascade are still unknown, we found *A. mellifera* cSP1 and cSPH42 are similar in sequence and domain structure to *M. sexta* PAP-1 and SPH-1, respectively. *M. sexta* PAP-1, SPH-1 and other clip-domain proteins participate in the proPO cleavage and activation (Tong *et al*., 2005; Zou & Jiang 2005). *A. mellifera* GB18313, 56% identical in amino acid sequence to *M. sexta* proPO-1, is the only *proPO* gene identified in the genome (Lourenco *et al*., 2005). Like most proPOs known so far, the honey bee *proPO* lacks a signal peptide and has the consensus sequence of NR^51^*F^52^G around the proteolytic activation site (*). These data suggest there is a conserved SP pathway to activate proPO in the bee.

### Serpins

SP inhibitors of the serpin superfamily are present in insect haemolymph to remove excess proteases and maintain homeostasis (Kanost, 1999). They are 45–55 kDa proteins with a conserved tertiary structure. Serpins regulate haemolymph coagulation, melanization and antimicrobial protein synthesis in arthropods. The reactive site loop near the carboxyl terminus is critical for inhibitory selectivity. Seven annotated genes in the honey bee genome encode five serpins and two serpin-like proteins with unusual insertions or extensions that may represent errors in gene prediction (Table 2). The ratio of SPs to serpins is 6.3 in *A. mellifera*, similar to that in *D. melanogaster* (5.3).

**Table 2:** Serine protease inhibitors (serpins) in *Apis mellifera*

		Homologous proteins					
						
Accession number	GenBank ID	Drosophila	Other arthropods^a^	Length (aa)	Signal peptide	Predicted reactive site	Target enzyme specificity^a^
serpin-1	GB17012	serpin-4	MsSerpin-1,2/AgSRPN-10	334	No	LR*RC	T
serpin-2	GB16472	serpin-4	MsSerpin-1,2	342	QG-ET	PL*SS	C
serpin-3	GB12279	spn-27 A	MsSerpin-3/AgSRPN-2	466	DG-KE	NK*NQ	T
serpin-4	GB13578	CG7219	AgSRPN-6	469	FG-QL	ER*DG	T
serpin-5	GB19582	serpin-5	MsSerpin-6	451	SA-QC	FR*SG	T
	GB10078^b^	CG14470		1543	VG-SP	ER*AE	T
	GB15070^b^	CG12807		612	YC-VD	ER*AG	T

a Ag, *Anopheles gambiae*; Ms, *Manduca sexta*; C, chymotrypsin; T, trypsin.

b GB10078 contains a carboxyl-terminal serpin domain; GB15070 contains a split serpin domain (maleszka3).

**Figure 4:**
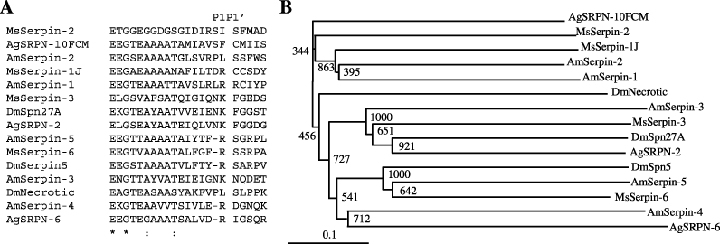
Sequence alignment and phylogenetic relationships of serpins from *Apis mellifera* and other insects. A. Amino acid sequence alignment of the P17-P4′ region. Identical residues are indicated by ‘*’, and similar residues by ‘:’. B. Phylogenetic tree based on alignment of full-length serpins selected from *A. mellifera, Anopheles gambiae*, *Drosophila melanogaster* and *Manducta sexta.*

While there is no experimental report on honey bee serpins, these inhibitors have been extensively investigated in moth, fly, and mosquito (Kanost *et al*., 2004). Through sequence alignment, we have identified putative orthologs of individual honey bee serpins and suggested their possible functions in the development and immunity (Fig. 4). *A. mellifera* serpin-1, −4 and −5 have an Arg at the predicted P1 site, the residue N-terminal to the cleavage site (Table 2), and *A. mellifera* serpin-3 has a Lys at the putative P1 position, suggesting that they may inhibit SPs with trypsin-like specificity. Consistent with the prediction that a few of the honey bee SPs are chymotrypsin-like (Table 1), one serpin (*A. mellifera* serpin-2) has a Leu at the putative P1 site.

We did not identify honey bee ortholog of Necrotic, a *Drosophila* serpin that controls the Toll pathway activation and spontaneous melanization. *A. mellifera* serpin-1 and −2 have a relatively high similarity (identity: 39%) to *M. sexta* serpin-1. *M. sexta* serpin gene-1 encodes 12 reactive site loop variants through alternative exon 9 usage (Kanost, 1999). Serpin-1 J blocks *proPO* activation by inhibiting PAP-1, −2 and −3 (Jiang *et al.*, 2003b). At a high concentration, *M. sexta* serpin-1I partly inhibited haemolymph protease 14, an initiation protease of the proPO activation cascade (Wang & Jiang, 2006). We identified *A. mellifera* serpin-3 (GB12279) as the ortholog of *D. melanogaster* Spn27A and *M. sexta* serpin-3, which inhibit PAPs to regulate melanization (Ligoxygakis *et al*., 2002b; Zhu *et al*., 2003). During embryonic development, Spn27A inhibits easter and suppresses activation of the Toll pathway that establishes the dorsoventral axis. The honey bee *serpin-5* (GB19582) may also be a negative regulator of melanization, since its ortholog *M. sexta* serpin-6 formed stable complexes with PAP-3 and HP8 (Zou & Jiang, 2005). Although experimental data are unavailable to support the proposed functions of the bee serpins, the observed sequence similarity provides useful working hypotheses to test.

### Gene expression

**Figure 5:**
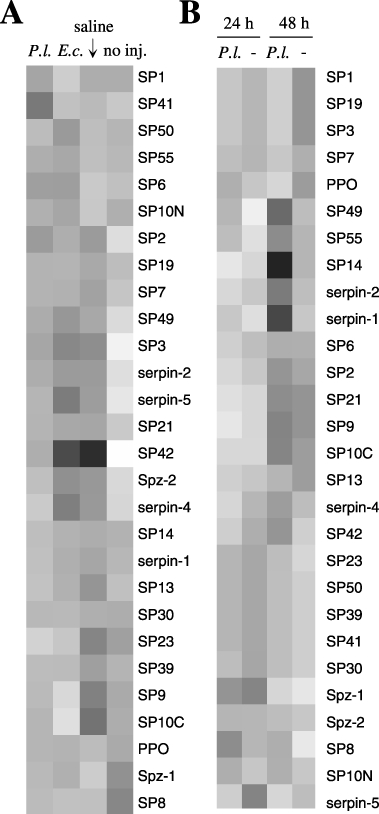
Quantitative RT-PCR analyses of *Apis mellifera* SP-related transcripts. A. RNA samples from adult workers at 24 h after injections of *Paenibacillus larvae* (*P.l.*), *E. coli* (*E.c.*), or saline and uninjected control. B. RNA from the 2nd instar larvae at 24 h or 48 h after feeding on the regular diet (–) or diet with an infective dose of *P. larvae*. Gene expression is shown in grey scale, with darker squares indicating higher expression levels. SP10N and SP10C: N- and C-terminal halves.

To investigate transcriptional regulation of the SP-related genes upon microbial infection, we injected adult workers with saline, *E. coli* or a honey bee pathogen (*Paenibacillus larva*). Real-time RT-PCR indicated that SP2, SP9, SP10 and SP23 mRNA levels increased after the saline injection (Fig. 5A). SP3, SPH42, SP49, serpin-2, serpin-4, serpin-5 and spätzle-2 transcripts were elevated after the saline or *E. coli* injection. We detected increases in the SP1, SP2, SP3, SP6, SP41, SPH42, SP49 and *serpin-2* transcript levels after the *P. larva* injection. Compared with the injection of saline or *E. coli*, the pathogen challenge gave rise to a much stronger induction of SP41 and SP6 gene transcription.

In contrast, mRNA level changes in the honey bee larvae were subtle at 24 h after the larvae fed on a diet containing *P. larva* spores (Fig. 5B). At 48 h, some SP and serpin transcripts became more abundant. Strong induction was observed for SP14, SPH42, SPH42, SPH55, serpin-1 and serpin-2 transcripts, whereas SP1, SP3, SP7, SPH19 and serpin-5 mRNA levels decreased. Perhaps, this pathogen evades the host defense (e.g. melanization) system by modulating the SP gene transcription.

## Conclusion

In this work, we explored the sequences and possible physiological functions of honey bee SPs/SPHs and serpins. Compared with *D. melanogaster* and *An. gambiae*, *A. mellifera* has much smaller families of SP, SPH, serpin, proPO and other immune proteins (Evans *et al*., 2006). Perhaps, defense strategies at the colony level largely alleviate the pressure on the immune system in individual insects, resulting in requirement for fewer genes functioning in defense against infection. Sequence, size, specificity and domain structure analyses of SPs provided useful clues to potential components of *A. mellifera* SP cascades. Quantitative RT-PCR indicated that many SPs and their regulators/substrates are immune responsive. Such information will be useful for elucidating the composition and function of SP-related protein systems in this social insect.

## Experimental procedures

### Database searching and sequence retrieving

*M. sexta* proPO-activating protease-1 (PAP-1) (Jiang *et al*., 1998) was used as a query to perform a BLASTP search of Official Gene Set-1 (Honey Bee Genome Sequencing Consortium, 2006) in the honey bee genome database, BeeBase (http://racerx00.tamu.edu/). Every tenth sequence from the primary list was retrieved and used as the query for another round of searching. The amino acid sequences encoded by predicted genes with significant Blast scores (*E*-value < 0.1) were retrieved and numbered in the order in which they were identified. Similarly, *M. sexta* serpin-1, serpin-3, serpin-6, proPO-1 and *D. melanogaster* spätzle sequences were used to search the database for homologous genes in *A. mellifera*.

### Sequence properties of *A. mellifera* SPs and SPHs

Sequences were categorized as SPs and SPHs by locating the conserved His, Asp, and Ser residues in the catalytic triad. If all three of these residues were present in the conserved TAAHC, DIAL and GDSGGP regions, the sequences were considered to be SPs. Sequences lacking one or more of these key residues were labelled SPHs. Protein sizes were calculated based on the entire predicted sequences.

### Identification of clip domains in SPs and SPHs

The retrieved *A. mellifera* SP and SPH sequences were reviewed manually to search for clip domains (Ross *et al*., 2003). SPs and SPHs containing regions N-terminal to the catalytic domain with six cysteine residues with Cys_5_ and Cys_6_ at adjacent positions were designated cSPs and cSPHs, respectively. For other SP-like proteins, domain organization and comparison were analysed by CDART at http://www.ncbi.nlm.nih.gov/, PROSITE at http://us.expasy.org/prosite, and SMART at http://smart.embl-heidelberg.de/smart. The chromosomal location and predicted exon-intron boundaries for each annotated sequence were acquired from BeeBase (Glean_3.gff).

### Multiple sequence alignment and phylogenetic analysis

SP catalytic domains and SPH protease-like domains were aligned using ClustalX (ftp://ftp-igbmc.u-strasbg.fr/pub/ClustalX/), and cladograms were constructed by the neighbour-joining method and displayed using Treeview (http://taxonomy.zoology.gla.ac.uk/rod/treeview.htm). A Blosum 30 matrix, with a gap penalty of 10 and an extension gap penalty of 0.1 were used in the multiple sequence alignment. In order to compare equivalent regions, 20 sequences lacking a significant portion of the protease-like domain were excluded from the analysis. SP catalytic domains from ∼50 residues upstream of the conserved His to ∼50 residues downstream of the reactive site Ser were compared. The corresponding region in SPHs was also included in the alignment. To compare the clip domain sequences, the region from one residue before Cys_1_ to one after Cys_6_ was analysed.

### Gene expression analysis

To screen for immune-related transcript changes, adult worker bees from a single local *A. mellifera* ligustica colony were injected with either phosphate-buffered saline or saline containing 10^3^ live *E. coli* cells or 10^3^ vegetative spores of *P. larvae* (Evans, 2004). These bees, along with the uninjected ones, were maintained at 34 °C and high humidity. To assess immune responses following a natural infection, eight 1st instar larvae from the same stock were given per os challenges of *P. larvae* in their diet (5 spores/ml), and then maintained at 34 °C and high humidity. Control larvae were fed on the same diet but without the spores. Following an incubation period, the adults and larvae were instantly frozen at −80 °C prior to RNA extraction. Total RNA was extracted from whole abdomens of the adults using Trizol (Invitrogen, Carlsbad, CA), whereas the larvae were extracted using the RNAqueous kit (Ambion, Austin, TX). After DNA removal, first-strand cDNA was synthesized as previously described (Evans, 2004).

Specific primer pairs (Table 3) with calculated annealing temperatures of 59.5–60.5 °C and expected product sizes of 150–200 bp were designed using Primer 3 (http://frodo.wi.mit.edu/cgi-bin/primer3/primer3_www.cgi). A total of 28 cDNAs for SP-related proteins were examined by real-time PCR. Each 25 µl reaction contained *Taq* DNA polymerase (1 U), 1 × buffer (Roche Applied Sciences), 1 mm dNTP mix, 2 mm MgCl_2_, 0.2 µm primers, 1 × SYBR-Green I dye (Applied Biosystems Foster City, CA), and 10 nm fluorescein. The thermal cycling conditions were 95 °C for 5 min and 40 cycles of 94 °C for 20 s, 60 °C for 30 s, 72 °C for 60 s and 78 °C for 20 s. Amplification was monitored on an iCycler (Bio-Rad, Hercules, CA). Primer pairs that caused dimer formation or other artifacts in no-template controls were excluded. The remaining pairs were arrayed randomly, in duplicate, across a 96-well plate, and all expression data were collected in parallel for each cDNA template. Thresholds were individually calculated for each target gene on the array. For adult bee samples, data were combined for the three replicates in each single-bee injection treatment (or control). The larval RNA samples were pooled before cDNA synthesis, and the cDNA was run in duplicate on the RT-PCR plate. Proper dissociation curves and correct product sizes were examined by melting curve analysis and agarose gel electrophoresis. The transcripts were normalized relative to the levels of ribosomal protein S5 (Evans, 2004; Evans & Wheeler, 2000). Transcript abundance values (Ct_control_– Ct_target_) for each gene were median-normalized across each panel of genes, clustered by average linkage clustering, and presented as relative grey-scale values using Eisen Cluster 3.0 and Eisen TreeView (http://rana.lbl.gov/EisenSoftware.htm).

**Table 3:** Oligonucleotides used in real time PCR of *Apis mellifera* SP-related genes

Locus	Forward Primer	Reverse primer	Gene ID
SP1	TGCTCATTGCGTTACATCGT	TTGTCAGCGCAAACAACTTC	GB16147
SP2	GCGTTTAGAAAGCGTTCGTC	TCCGCGCAAAGTAAGCTATT	GB14247
SP3	ATGGACCCTTGTTACCACCA	GTTGCGAAGGGTTCAAAGAA	GB11698
SP6	CGATGACGATGACATTCCTG	TGTGTCCACCCACGATTCTA	GB14077
SP7	GGCTGGGTTCTTGGTGTTTA	GCTCGACTGTGGTGTAACGA	GB17145
SP8	GTTTGGTCGACGGAAGAAAA	CCGTCGACTCGAAATCGTAT	GB18767
SP9	GAGATGTTGAATGGCACGAA	CCACCACTATCTCCCTGACAA	GB18732
SP10N	CCGGTGAACTTGGAAAAGAT	CTTCGCCAGGAATAATGGAA	GB17927
SP10C	GAGATGTTGAATGGCACGAA	CCACCACTATCTCCCTGACAA	GB17927
SP13	CGGAGCTTAAATGCGAAGAA	TTGTTCCTAGAGCAACCATGTG	GB15640
SP14	GATTACCCAATGGCATCGAC	GCTGGTGAACCGCAAGTATT	GB14044
SPH19	ACCATCGAGAAAACCACGAC	GTACACGCTTTCCGTTGGAT	GB17345
SP21	GCCGGAAACTTACACGGTTA	CGATAATGTGCTTGCGGTAA	GB16220
SP23	AACGGAAACGAAATGGACAG	GAGCACATGCTTGAACGAAA	GB12538
SP30	CACCAGAAGGCACTCTCACA	CCTGAGCGAAGCCTAAATTG	GB19649
SPH39	GCGCCAGGAAACTCTGTTAG	ACGAAGCTTCCCCGTTTATT	GB14366
SPH41	ACCGGCACAAGCAAAATTAC	GCGAACTCTTCGTGTTGTCA	GB10943
SPH42	GAAGTCCCCTTGTTTGTCCA	TCGATCCAATCACGAACAGA	GB11298
SP49	TGTGATGGCATAGCAGATTGT	CAGGCACCATAATCACAACG	GB15317
SPH50	GCAAATCGAAAGGGAAATGA	CTGATGGAAAGCTGGTGGTT	GB14001
SPH55	GTCAACGACGTGGAAGGAAT	CGTTGGAAGACATCCCGTAT	GB13397
serpin-1	CATGGTGACATGCCAATGTT	CGAGTTGTATTTGCAAGCATTT	GB17012
serpin-2	TCCATGGAGGCAGCAAATA	CCATTGGCCTTTAAAATAAACTG	GB16472
serpin-3	CGGGAGACGAAACTGATGAT	TTCACCTTGAGCTCCTTCGT	GB12279
serpin-4	CTGGGCCACGTGTAGATTTT	ATGTCCATTGCTGCTTTTCC	GB13578
serpin-5	ACTCAGCGAACCGATTATGG	GGACAGCATTTGGATTCGTT	GB19582
Spz-1	TGCACAAATTGTTTTTCCTGA	GTCGTCCATGAAATCGATCC	GB15688
Spz-2	AATCGAAGGTTTCGCTGAAG	TTCCGGTATTATGGAACCATTT	GB13503
PPO	AGATGGCATGCATTTGTTGA	CCACGCTCGTCTTCTTTAGG	GB18313

## Abbreviations

SP and cSP: serine protease and clip-domain serine protease
cSPH and SPH: serine protease homolog with and without clip domain
PO and proPO: phenoloxidase and its precursor
PAP: proPO-activating protease
HP: haemolymph protease
RT-PCR: reverse transcriptase-polymerase chain reaction
